# Antidepressant-Like Effect of the Endogenous Neuroprotective Amine, 1MeTIQ in Clonidine-Induced Depression: Behavioral and Neurochemical Studies in Rats

**DOI:** 10.1007/s12640-017-9715-z

**Published:** 2017-04-03

**Authors:** Lucyna Antkiewicz-Michaluk, Irena Romańska, Agnieszka Wąsik, Jerzy Michaluk

**Affiliations:** Department of Neurochemistry, Institute of Pharmacology Polish Academy of Sciences, 12 Smętna Street, 31-343 Kraków, Poland

**Keywords:** Clonidine model of depression, Forced swim test, HPLC, Metabolism of monoamines, Brain structures, Rat

## Abstract

Biogenic amines such as norepinephrine, dopamine, and serotonin play a well-described role in the treatment of mood disorders especially depression. Animal models are widely used to study antidepressant-like effect in rodents; however, it should be taken into account that pharmacological models do not always answer to the complexity of the disease processes. This study verified the behavioral (forced swim test (FST), locomotor activity test) and neurochemical effects (monoamines metabolism) of a low dose of clonidine (0.1 mg/kg i.p.) which was used as an experimental model of depression. In such pharmacological model, we investigated the antidepressant-like effect of an endogenous neuroprotective amine, 1-methyl-1,2,3,4-tetrahydroisoquinoline (1MeTIQ) administered in a dose of 25 mg/kg (i.p.) before clonidine in the behavioral and neurochemical tests carried out in rats. The behavioral study has shown that clonidine produced depression in the locomotor activity test but did not cause pro-depressive effect in the FST. 1MeTIQ produced antidepressant-like effect in the FST and completely antagonized clonidine-induced sedation in the locomotor activity test. Neurochemical data demonstrated that clonidine produced a significant inhibition of monoamine metabolism in the central nervous system. The release of dopamine, noradrenaline, and serotonin as well as the rate of their metabolism were diminished in the investigated brain structures (frontal cortex, hypothalamus, and striatum). 1MeTIQ completely antagonized the clonidine-induced depression of monoaminergic systems and restored their levels to the control values. 1MeTIQ as an endogenous neuroprotective compound with a distinct antidepressant-like activity in rodents produces hope on the efficiency of antidepressant medicines for future practical clinical use.

## Introduction

Animal models are widely used to study antidepressant-like effect in rodents. However, it should be mentioned that pharmacological models do not always take into account the complexity of the disease process which is thought to be multifactorial. What is more, despite intensive research conducted in many scientific centers worldwide, the mechanism evoking depression is not clear till now. It is well known that monoamine neurotransmitters, such as dopamine (DA), noradrenaline (NA), and serotonin (5-HT) in the central nervous system play a key role in the pathophysiology of depression (Mayeux et al. 1984; Chan-Palay and Asan 1989; Elhwuegi 2004). All commercially available antidepressants, including the tricyclics, monoamine oxidase (MAO) inhibitors, non-selective adrenoceptor antagonists, and selective 5-HT reuptake inhibitors (SSRIs) work via various mechanisms to increase synaptic concentrations of monoamines (Cryan and Lucki 2000; Mico et al. 2006; Elhwuegi 2004). The monoaminergic hypothesis claims that depression is caused by a decreased monoaminergic function of the brain. The vast body of available world literature has shown that antidepressants act mostly on the 5-HT and NA systems (Papakostas et al. 2007). DA has been classically associated with abuse disorders and psychosis, but it may play also a role in depression. The 5-HT, NA, and DA systems are linked by complex interactions, thus most of the effects exerted on 5-HT or NA systems affect DA release (Westenberg 1999; Invernizzi and Garattini 2004).

However, abnormalities in monoaminergic neurotransmission are associated with a number of neurological disorders, like Parkinson’s disease, and as generally accepted, also psychiatric disorders, like schizophrenia or depression. Although the mechanism provoking depression has not been clearly elucidated, however, oxidative stress associated with generation of reactive oxygen species (ROS) can be one of the main causes in molecular processes underlying this disease. Recently, it was shown that enhanced neurodegeneration associated with depression may be partially attributed to oxidative stress and inflammation (Maes 2008; Maes et al. 2011; Makhija and Karunakaran 2013). Therefore, other strategies beyond monoamine (DA, NA, and 5-HT) reuptake inhibition such as anti-oxidative agents may represent promising antidepressant therapies. For instance, many anti-oxidant agents have shown antidepressant-like activity in animal models of depression (Lopresti et al. 2012; El-Naga et al. 2014; Tizabi et al. 2014).

In the present study, we analyzed the antidepressant potential of the endogenous amine from the tetrahydroisoquinoline group, 1-methyl-1,2,3,4-tetrahydroisoquinoline (1MeTIQ) in the clonidine model of depression. Our previous studies demonstrated antidepressant-like effect of 1MeTIQ in different animal models of depression (Antkiewicz-Michaluk et al. 2014b; Możdżeń et al. 2014; Wąsik et al. 2013). 1MeTIQ has a special position among new investigated substances with antidepressant-like effect because it is a neuroprotective compound with an anti-parkinsonian potential (Antkiewicz-Michaluk et al. 2003, 2004, 2014a; Makino et al. 1990; Kotake et al. 1995; Tasaki et al. 1991). Additionally, 1MeTIQ reversibly inhibited MAO A and B activities at micro-molar concentrations (Patsenka and Antkiewicz-Michaluk 2004) and showed the ability to inhibit free radical formation and to abolish H_2_O_2_ generation from dopamine via the Fenton reaction (Antkiewicz-Michaluk et al. 2006). 1MeTIQ which is present in the brain, is a mixture of (R)- and (S)-enantiomers enzymatically synthesized from 2-phenylethylamine and pyruvate by the 1MeTIQ-synthesizing enzyme, a membrane-bound protein localized in the mitochondrial synaptosomal fraction (Yamakawa and Ohta 1997; Yamakawa et al. 1999). 1MeTIQ was shown to act as an anti-dopaminergic agent, but in contrast to typical neuroleptics, it did not induce catalepsy in animals (Antkiewicz-Michaluk et al. 2000, 2014a). In functional studies, 1MeTIQ inhibited an apomorphine-induced hyperactivity at doses where it had no effect on the spontaneous locomotor activity of rats (Antkiewicz-Michaluk et al. 2001). In addition, in vitro as well as in vivo studies have demonstrated that 1MeTIQ behaves as a partial dopamine agonist and monoamine reuptake inhibitor (Antkiewicz-Michaluk et al. 2007; Patsenka et al. 2004).

Presently, we examined behavioral and neurochemical effects of a low-dose clonidine (0.1 mg/kg i.p.), and then evaluated antidepressant-like effect of 1MeTIQ in the clonidine model of depression. Clonidine is an alpha_2_ adrenoceptor agonist used in the treatment of hypertension. Clonidine has been shown to produce depressive behavioral effects in laboratory animals, and thus it has been suggested to be a suitable animal model of depression (Enginar and Eroğlu 1990; Lim et al. 2016; Parale and Kulkarni 1986; Park et al. 2016).

Behavioral forced swim test (FST) was used to examine the antidepressant properties of 1MeTIQ. The FST is a test with high predictive validity for antidepressant efficacy in human depression. Recently, a behavior-sampling technique was developed that scores individual response categories, including immobility, swimming, and climbing (Detke et al. 1995). Selective serotonin reuptake inhibitors increase swimming behavior while drugs acting primarily to elevate extracellular levels of NA or DA increase climbing behavior (Borsini 1995; Detke et al. 1995; Detke and Lucki 1996; Nixon et al. 1994). Additionally, the locomotor activity test was used to check motor function of rats in the clonidine model of depression.

In the second part of the study, in addition to the behavioral tests, we also carried out neurochemical ex vivo studies to determine the tissue concentration of monoamines as well as the rate of their metabolism in rat brain structures (frontal cortex, hypothalamus, and striatum).

## Materials and Methods

### Animals

The experiments were carried out on male Wistar rats (Charles River) with an initial body weight of 230–240 g (about 7 weeks old). The animals were kept under standard laboratory conditions with free access to laboratory food and tap water, at room temperature of 22 °C with an artificial day-night cycle (12/12 h, lights on at 7 a.m.). All the procedures were carried out in accordance with the National Institutes of Health Guide for the Care and Use of Laboratory Animals and were granted an approval from the Bioethics Commission as compliant with Polish Law. The experimental protocols were approved by the Local Bioethics Commission of the Institute of Pharmacology, Polish Academy of Sciences in Kraków, Poland.

### Drugs

1MeTIQ was synthesized in the Department of Drug Chemistry, Institute of Pharmacology, Polish Academy of Sciences, Krakow, Poland; the purity of the compound was verified by measurement of the melting point, and homogeneity was assessed on a chromatographic column. Clonidine (hydrochloride) was from Sigma-Aldrich (St. Louis, MO, USA). The drugs were dissolved in sterile 0.9% NaCl solution and were injected in a volume of 4 ml/kg.

### Behavioral Experiments

#### The Forced Swim Test Procedure

The studies were carried out on rats and were based on the method of Porsolt et al. (1978). All the animals were individually tested in the FST on two consecutive days with 1 session/day. On the first day, the rats were individually placed in non-transparent plastic cylinders (diameter, 23 cm; height, 50 cm) containing water of a height of 30 cm, maintained at 25–26 °C. They were allowed to swim for 15 min before being removed (pre-test session). After, the animals were dried and returned to their home cages. The procedure was repeated 24 h later, and the time of the escape-oriented behavior of the rats was recorded (for 5-min test session). The observed behavioral parameters (in the order of priority) were time spent floating in water (*immobility*), *swimming*, and struggling (*climbing*). According to Detke et al. (1995), the immobility is described as the behavior of the rat when it makes only the movements necessary to keep its head above the water. In this case, animals can make certain slight swimming movements in order to remain afloat. Climbing is defined as vigorous movements of four limbs, with the front paws breaking against the wall of the cylinder. During swimming, rats make coordinated and sustained movements (more than necessary) with all four limbs, usually traveling around the interior of the cylinder but without breaking the surface of the water with forelimbs. Water was changed between subjects. The FST was performed 120 min after 1MeTIQ (25 mg/kg i.p.) and 60 min after clonidine (0.1 mg/kg i.p.) administration. In the combined treatment groups, 1MeTIQ was administered 60 min before clonidine injection. Six to eight animals per group were used. The experiments were carried out between 9 a.m. and 3 p.m.

#### Locomotor Activity

The locomotor activity was measured in actometers (Opto-Varimex activity monitors, Columbus Instruments, USA) linked on-line to an IBM-PC-compatible computer. Each cage (43 × 44 × 25 cm) was surrounded with a 15 × 15 array of photocell beams located 3 cm from the floor surface. Interruptions of these photocell beams were counted as a measure of horizontal locomotor activity which was defined as the traveled distance (cm). Locomotor activity was analyzed using Auto-Track Software Program (Columbus Instruments, USA). The horizontal locomotor activity was measured 30 min after the administration of clonidine (0.1 mg/kg i.p.) and 60 min after 1MeTIQ (25 mg/kg i.p.) during 120 min. In the combined group, 1MeTIQ was administered 30 min before clonidine. Six to eight animals per group were used.

### Biochemical Studies

#### Ex Vivo: Monoamine Metabolism in Rat Brain Structures

Immediately after the end of the behavioral test (FST), the rats were killed by decapitation and the brain was rapidly removed and dissected into different brain structures (*frontal cortex*, *hypothalamus*, and *striatum*) on an ice-cold glass plate. The structures were frozen on solid CO_2_ (−70 °C) until used for biochemical assays. DA and its metabolites, the intra-neuronal, 3,4-dihydroxyphenylacetic acid (DOPAC); the extraneuronal, 3-methoxytyramine (3-MT) and final metabolite, homovanillic acid (HVA); and NA and its main extraneuronal brain metabolite, normetanephrine (NM) and 5-HT and its intra-neuronal metabolite 5-hydroxyindolacetic acid (5-HIAA) were assayed by means of high-performance liquid chromatography (HPLC) with electrochemical detection (ED). The tissue samples were weighted and homogenized in ice-cold 0.1 M trichloroacetic acid containing 0.05 mM ascorbic acid. After centrifugation (10,000×*g*, 5 min), the supernatants were filtered through RC58 at 0.2 μm cellulose membranes (Bioanalytical Systems, West Lafayette, IN, USA). The chromatograph HP 1050 (Hewlett-Packard, Golden, CO, USA) was equipped with Hypersil column BDS-C18 (4 × 100 mm, 3 μm). The mobile phase consisted of 0.05 M citrate-phosphate buffer at pH 3.5, 0.1 mM EDTA, 1 mM sodium octyl sulfonate, and 3.5% methanol. The flow rate was maintained at 1 ml/min. DA, NA, and 5-HT and their metabolites were quantified by peak area comparisons with standards run on the day of analysis (*ChemStation, Hewlett-Packard software computer program*).

### Calculations and Statistics

The results of the behavioral and biochemical studies were analyzed by a two-way analysis of variance (ANOVA) followed when appropriate, by Duncan’s post hoc test. The data were considered statistically significant when *P <* 0.05.

The total catabolism rate for dopamine was assessed from the ratio of the final dopamine metabolite concentration HVA to dopamine concentration and expressed as the catabolic rate index *(HVA)/(DA) × 100*; the index of dopamine release as the ratio: *(3-MT)/(DA) × 100*; and the factor of dopamine reuptake inhibition as the ratio *(3-MT)/(DOPAC) × 100*. Analogously, the rate of noradrenaline metabolism was expressed as the ratio of the extraneuronal metabolite, normetanephrine to noradrenaline: *(NM)/(NA) × 100* and serotonin as the ratio: *(5-HIAA)/(5-HT) × 100*. The indices were calculated using concentrations from individual tissue samples (Antkiewicz-Michaluk et al., 2001).

## Results

### Behavioral Experiments

#### The Effects of Clonidine and 1MeTIQ in the FST

**Fig. 1:**
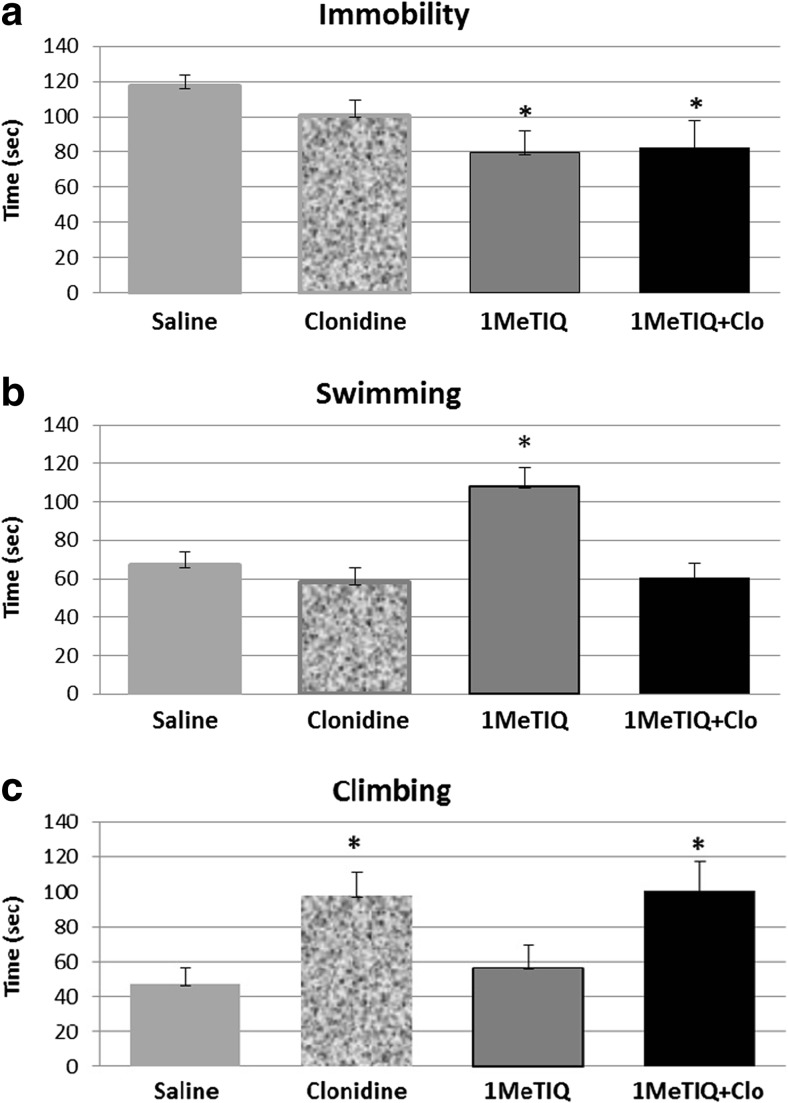
The effect of clonidine, 1MeTIQ, and their combined treatment in FST. The rats received a single injection of saline (control group); 1MeTIQ (25 mg/kg i.p.) was administered 1 h before clonidine (0.1 mg/kg i.p.) in the joint group, and 1 h after clonidine administration, the FST was carried out. The rats were placed into the cylinder for 5 min, and during this time, three types of behavior were measured: immobility (**a**), swimming (**b**), and climbing (**c**). The data are means ± SEM, the number of animals per group (*n* = 6–8). *P < 0.05 difference from the control group (SAL); ^+^ *P* < 0.05 difference from the clonidine group with Duncan’s test

Clonidine (0.1 mg/kg, i.p.) did not produce pro-depressive activity in the FST and did not change the immobility time *F*_(1, 27)_ = 3.31; *P* < 0.0987 (Fig. 1a); however, it significantly enhanced the climbing behavior *F*_(1, 27)_ = 12.24; *P* < 0.0016 (Fig. 1c) and slightly decreased the swimming time in rats (Fig. 1b). 1MeTIQ (25 mg/kg, i.p.) administration produced antidepressant-like effect and significantly reduced (by 35%; *F*_(1, 27)_ = 4.89; *P* < 0.0487) the immobility time in the FST (Fig. 1a). The two-way ANOVA showed a significant increase in the swimming activity after 1MeTIQ (*F*_(1, 27)_ = 8.16; *P* < 0.0081) and no change in the climbing activity (*F*_(1, 27)_ = 0.16; ns).

Duncan’s post hoc test demonstrated that combined treatment with 1MeTIQ + clonidine significantly increased (by 90%, *P* < 0.0159) the climbing activity in the FST (Fig. 1c).

#### The Effect of 1MeTIQ on Clonidine-Produced Depression in the Locomotor Activity Test

**Fig. 2:**
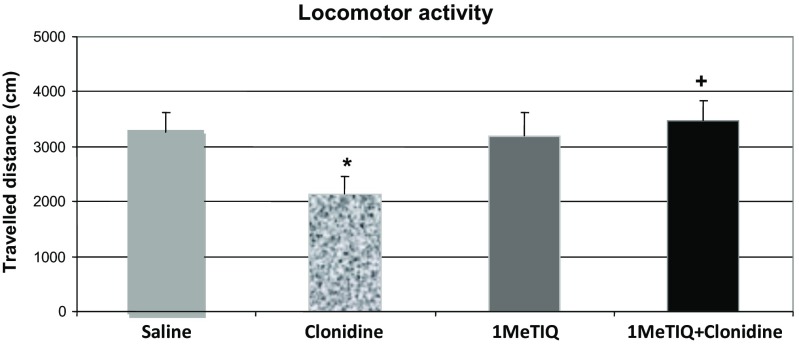
The effect of 1MeTIQ on the clonidine induced a locomotor depression in rats. The rats received a single injection of saline (control group), 1MeTIQ (25 mg/kg i.p.), and clonidine (0.1 mg/kg i.p.); in combined group, 1MeTIQ was administered 30 min before clonidine, and 30 min after clonidine administration, the rats were placed into actometers for 120 min. The data are means ± SEM, the number of animals per group (*n* = 6–8). *P < 0.05 difference from the control group (SAL); ^+^ *P* < 0.05 difference from the clonidine group with Duncan’s test

The results of the locomotor activity test showed a significant inhibitory effect of clonidine (0.1 mg/kg i.p.). The two-way ANOVA revealed a significant effect of clonidine (*F*_(1, 21)_ = 15.11; *P* < 0.0008) and its interaction with 1MeTIQ (*F*_(1, 21)_ = 5.87; *P* < 0.0244) in the locomotor activity test. Duncan’s post hoc test demonstrated that clonidine decreased locomotor activity by 35% when compared with the saline group (*P* < 0.0004). 1MeTIQ alone did not affect (*F*_(1, 21)_ = 0.18; ns) the locomotor activity but significantly (*P* < 0.05) antagonized the clonidine-induced depression in rats (Fig. 2).

### Neurochemical Studies

#### The Effect of 1MeTIQ on the Dopamine Metabolism in Rat Brain Structures in the Clonidine-Produced Model of Depression

##### Dopamine

The two-way ANOVA indicated a significant effect of 1MeTIQ treatment on DA concentration only in the frontal cortex (*F*_(1, 23)_ = 20.31, *P* < 0.0001) and no significant effect of clonidine and interaction of 1MeTIQ + CLO. Duncan’s post hoc test revealed a significant increase in DA concentration in the 1MeTIQ-treated group (about 28% of control saline group, *P* < 0.05) and in the combined group 1MeTIQ + CLO (about 60% of control, *P* < 0.01) (Table 1).

**Table 1:** Duncan’s post hoc test revealing a significant increase in DA concentration in the 1MeTIQ-treated group and in the combined group 1MeTIQ + CLO

Treatments		Number	DA (ng/mg prot.)	DOPAC (ng/mg prot.)	3-MT (ng/mg prot.)	HVA (ng/mg prot.)
T1	T2					
Frontal cortex						
Saline	Saline	6	565 ± 38	118 ± 6.0	16 ± 1.8	136 ± 5.9
Saline	CLO/0,1	8	634 ± 56	110 ± 9.1	9 ± 1.2*	125 ± 9.2
1MeTIQ/25	Saline	6	725 ± 63*	90 ± 8.2*	31 ± 2.0**	135 ± 11.2
1MeTIQ/25	CLO/0,1	8	914 ± 31**^++^	92 ± 2.8*	29 ± 2.3**^++^	152 ± 9.1
Effect of 1MeTIQ			*F*_(1, 23)_ = 20.31	*F*_(1, 23)_ = 10.54	*F*_(1, 23)_ = 88.37	*F*_(1, 23)_ = 1.98
			*P* < 0.0001	*P* < 0.003	*P* < 0.0000001	ns
Effect of CLO			*F*_(1, 23)_ = 7.01	*F*_(1, 23)_ = 0.14	*F*_(1, 23)_ = 5.79	*F*_(1, 23)_ = 0.10
			*P* < 0.01	ns	*P* < 0.02	ns
Interaction of 1MeTIQ + CLO			*F*_(1, 23)_ = 1.50	*F*_(1, 23)_ = 0.41	*F*_(1, 23)_ = 1.65	*F*_(1, 23)_ = 2.35
			ns	ns	ns	ns
Hypothalamus						
Saline	Saline	6	549 ± 53	101 ± 11	11 ± 1	66 ± 8
Saline	CLO/0,1	8	592 ± 28	81 ± 5*	5 ± 1**	48 ± 6
1MeTIQ/25	Saline	6	620 ± 49	59 ± 8**^, ++^	18 ± 1**^, ++^	39 ± 6**
1MeTIQ/25	CLO/0,1	8	649 ± 33	45 ± 4**^, ++^	13 ± 2^++, ^^^	40 ± 5**
Effect of 1MeTIQ			*F*_(1, 23)_ = 2.57	*F*_(1, 23)_ = 31.23	*F*_(1, 23)_ = 28.93	*F*_(1, 23)_ = 7.29
			ns	*P* < 0.000011	*P* < 0.00001	*P* < 0.012771
Effect of CLO			*F*_(1, 23)_ = 0.79	*F*_(1, 23)_ = 5.61	*F*_(1, 23)_ = 15.99	*F*_(1, 23)_ = 1.56
			ns	*P* < 0.026548	*P* < 0.000564	ns
Interaction of 1MeTIQ + CLO			*F*_(1, 23)_ = 0.029	*F*_(1, 23)_ = 0.1945	*F*_(1, 23)_ = 0.06	*F*_(1, 23)_ = 2.2699
			ns	ns	ns	ns
Striatum						
Saline	Saline	6	10,848 ± 152	1950 ± 63	213 ± 18	883 ± 52
Saline	CLO/0,1	8	11,090 ± 293	1853 ± 57	116 ± 14**	759 ± 56
1MeTIQ/25	Saline	6	11,714 ± 452	1398 ± 121**	208 ± 9	726 ± 101
1MeTIQ/25	CLO/0,1	8	11,678 ± 759	1272 ± 83**^, ++^	174 ± 23^+^	733 ± 69
Effect of 1MeTIQ			*F*_(1, 23)_ = 1.89	*F*_(1, 23)_ = 57.57	*F*_(1, 23)_ = 1.97	*F*_(1, 23)_ = 1.71
			ns	*P* < 0.0000001	ns	ns
Effect of CLO			*F*_(1, 23)_ = 0.04	*F*_(1, 23)_ = 3.93	*F*_(1, 23)_ = 11.99	*F*_(1, 23)_ = 0.69
			ns	ns	*P* < 0.002	ns
Interaction of 1MeTIQ + CLO			*F*_(1, 23)_ = 0.07	*F*_(1, 23)_ = 0.19	*F*_(1, 23)_ = 2.82	*F*_(1, 23)_ = 0.88
			ns	ns	ns	ns

1MeTIQ in a dose of 25 mg/kg i.p. was administered 30 min before clonidine (0.1 mg/kg i.p.; combined group). The control group received saline. Animals were decapitated 90 min after clonidine injection. The concentration of dopamine (DA) and its metabolites was expressed as nanograms per gram of wet tissue. The data are the means ± SEM. The results were analyzed by means of two-way ANOVA analysis of variance, followed when appropriate, by post hoc Duncan’s test. Statistical significance

*P < 0.05; **P < 0.01 vs. control group; ^***+***^ *P* < 0.05; ^*++*^ *P* < 0.01 vs. clonidine-treated group—statistical significance; ^^*P* <0.01 vs. 1MeTIQ-treated group

##### Dopac

The two-way ANOVA showed a significant effect of 1MeTIQ on DOPAC level in all investigated structures (the frontal cortex *F*_(1, 23)_ = 10.54, *P* < 0.003; hypothalamus *F*_(1, 23)_ = 31.23, *P* < 0.000001; striatum *F*_(1, 23)_ = 57.57, *P* < 0.00000) while clonidine affected it only in the hypothalamus (*F*_(1, 23)_ = 5.61, *P* < 0.0265), and no significant effect of interaction of these drugs was observed. Duncan’s post hoc test revealed a significant decrease in DOPAC concentration after these treatments (from 20 to 40% of the control values), most strongly pronounced after 1MeTIQ in the hypothalamus (Table 1).

##### 3-MT

The two-way ANOVA demonstrated a significant effect of 1MeTIQ on the concentration of an extraneuronal metabolite of dopamine, 3-MT in the frontal cortex (*F*_(1, 23)_ = 88.37, *P* < 0.00000) and hypothalamus (*F*_(1, 23)_ = 28.93, *P* < 0.00001) and no significant effect of interaction of 1MeTIQ + CLO. The ANOVA showed also a significant effect of clonidine on 3-MT in all analyzed structures (the frontal cortex *F*_(1, 23)_ = 5.79, *P* < 0.025; hypothalamus *F*_(1, 23)_ = 15.99, *P* < 0.000564; striatum *F*_(1, 23)_ = 11.99, *P* < 0.002). As shown by Duncan’s post hoc test, 1MeTIQ significantly increased the level of 3-MT in the frontal cortex and hypothalamus, in contrast, clonidine significantly decreased its concentration in all structures (Table 1).

##### HVA

The two-way ANOVA demonstrated a significant effect of 1MeTIQ only in the hypothalamus (*F*_(1, 23)_ = 7.29, *P* < 0.0127) and no effect of clonidine and the interaction of 1MeTIQ with clonidine in these structures. Duncan’s post hoc test revealed a significant decrease (*P* < 0.01) in the HVA level after treatment with 1MeTIQ alone and as well as in the combined group 1MeTIQ + clonidine (Table 1).

##### The Indices of Dopamine Catabolism

The two-way ANOVA indicated a significant effect of the treatments: 1MeTIQ, clonidine, and their interaction 1MeTIQ + clonidine on the rate of total dopamine catabolism (HVA)/(DA). The effect of 1MeTIQ treatment was significant in all investigated structures: frontal cortex (*F*_(1, 23)_ = 6.440, *P* < 0.010), hypothalamus (*F*_(1, 23)_ = 23.78, *P* < 0.00006), and striatum (*F*_(1, 23)_ = 5.19, *P* < 0.030), while clonidine showed a significant effect only in the hypothalamus (*F*_(1, 23)_ = 5.46, *P* < 0.028), and their interaction was significant also in these structures (*F*_(1, 23)_ = 5.95, *P* < 0.022). Duncan’s post hoc test demonstrated a significant decrease in the rate of total dopamine metabolism with the same direction after treatment of 1MeTIQ and clonidine (Table 2).

**Table 2:** Duncan’s post hoc test demonstrating a significant decrease in the rate of total dopamine metabolism

Treatments		Number	(HVA)/(DA)	(3-MT)/(DA)	(3-MT)/(DOPAC)
T1	T2				
Frontal cortex					
Saline	Saline	6	25 ± 2.5	3 ± 0.3	14 ± 1.5
Saline	CLO/0,1	8	20 ± 1.5	1 ± 0.2**	8 ± 1.3*
1MeTIQ/25	Saline	6	19 ± 2.1*	4 ± 0.3**	36 ± 4.2**
1MeTIQ/25	CLO/0,1	8	17 ± 1.2**	3 ± 0.2^++^	32 ± 2.2**^, ++^
Effect of 1MeTIQ			*F*_(1, 23)_ = 6.44	*F*_(1, 23)_ = 36.38	*F*_(1, 23)_ = 99.18
			*P* < 0.01	*P* < 0.000004	*P* < 0.0000001
Effect of CLO			*F*_(1, 23)_ = 3.59	*F*_(1, 23)_ = 21.54	*F*_(1, 23)_ = 4.11
			ns	*P* < 0001	*P* < 0.05
Interaction of 1MeTIQ + CLO			*F*_(1, 23)_ = 0.45	*F*_(1, 23)_ = 0.06	*F*_(1, 23)_ = 0.04
			ns	ns	ns
Hypothalamus					
Saline	Saline	6	12 ± 0.8	2 ± 0.3	11 ± 2
Saline	CLO/0,1	8	8 ± 0.9**	1 ± 0.1**	6 ± 0.8*
1MeTIQ/25	Saline	6	6 ± 0.4**	3 ± 0.1**^, ++^	31 ± 2**^, ++^
1MeTIQ/25	CLO/0,1	8	6 ± 0.6**	2 ± 0.3^++^	28 ± 4**^++^
effect of TIQ			*F*_(1, 23)_ = 23.78	*F*_(1, 23)_ = 17.65	*F*_(1, 23)_ = 59.00
			*P* < 0.000063	*P* < 0.000341	*P* < 0.0000001
Effect of CLO			*F*_(1, 23)_ = 5.46	*F*_(1, 23)_ = 21.34	*F*_(1, 23)_ = 4.05
			*P* < 0.028484	*P* < 0.00012	*P* < 0.05
interaction of 1Me TIQ + CLO			*F*_(1, 23)_ = 2.95	*F*_(1, 23)_ = 0.29	*F*_(1, 23)_ = 0.15
			ns	ns	ns
Striatum					
Saline	Saline	6	8 ± 0.5	2.0 ± 0.2	11 ± 1.0
Saline	CLO/0,1	8	7 ± 0.5	1.0 ± 0.1**	6 ± 0.6*
1MeTIQ/25	Saline	6	6 ± 0.8*	1.8 ± 0.1	15.1 ± 0.8*
1MeTIQ/25	CLO/0,1	8	6 ± 0.5*	1.5 ± 0.2	14 ± 1.5*^, ++^
Effect of 1MeTIQ			*F*_(1, 23)_ = 5.19	*F*_(1, 23)_ = 0.70	*F*_(1, 23)_ = 29.13
			*P* < 0.03	ns	*P* < 0.00001
Effect of CLO			*F*_(1, 23)_ = 1.24	*F*_(1, 23)_ = 13.62	*F*_(1, 23)_ = 6.46
			ns	*P* < 0.001	*P* < 0.01
Interaction of 1MeTIQ + CLO			*F*_(1, 23)_ = 1.68	*F*_(1, 23)_ = 4.08	*F*_(1, 23)_ = 1.68
			ns	ns	ns

1MeTIQ in a dose of 25 mg/kg i.p. was administered 30 min before clonidine (0.1 mg/kg i.p.; combined group). The control group received saline. Animals were decapitated 90 min after clonidine injection. The rate of DA metabolism was expressed as the ratio of its metabolite concentrations to dopamine, (metabolites)/to (DA) × 100. The indices were calculated using concentrations from individual tissue samples. The data are the means ± SEM. The results were analyzed by means of two-way ANOVA analysis of variance, followed when appropriate, by post hoc Duncan’s test

*P < 0.05; **P < 0.01 vs. control group; ^***+***^ *P* < 0.05; ^*++*^ *P* < 0.01 vs. clonidine-treated group—statistical significance

At the same time, the statistical analysis demonstrated a significant increase in the rate of dopamine release measured as the ratio of (3-MT)/(DA) after 1MeTIQ treatment in the frontal cortex and hypothalamus (*F*_(1, 23)_ = 36.38, *P* < 0.000004; *F*_(1, 23)_ = 17.65, *P* < 0.000341, respectively), but opposite effect was observed after clonidine injection, i.e., a significant decrease of the rate of dopamine release in all investigated structures (Table 2).

The two-way ANOVA indicated a significant but opposite effects of the 1MeTIQ and clonidine treatments on the factor of dopamine reuptake inhibition calculated as the ratio (3-MT)/(DOPAC). Duncan’s post hoc test demonstrated its significant increase (about 200% of the control value; *P* < 0.001) after 1MeTIQ administration but a significant decrease (about 50% of the control value; *P* < 0.01) after clonidine injection in rat brain structures which shows the clear inhibitory effect of 1MeTIQ on the dopamine transporter (DAT) expressed with the index (3-MT)/(DOPAC) (Table 2).

#### The Effect of 1MeTIQ on Clonidine-Produced Depression Manifested by Changes in the Metabolism of Noradrenaline in Rat Brain Structures

##### Noradrenaline

One-way ANOVA revealed a significant effect of clonidine (*F*_(1, 23)_ = 20.21, *P* < 0.0001) in the frontal cortex and 1MeTIQ in the frontal cortex and striatum (*F*_(1, 23)_ = 5.62, *P* < 0.02; *F*_(1, 23)_ = 6.705, *P* < 0.01, respectively). Duncan’s post hoc test indicated a significant increase in the concentration of noradrenaline after clonidine (by about 28% of the control group; *P* < 0.05) and in the combined group 1MeTIQ + clonidine (about 60% of the control group, *P* < 0.001) in the frontal cortex as well as in the striatum (about 36% of the control group; *P* < 0.05) (Table 3).

**Table 3:** Duncan’s post hoc test indicating a significant increase in the concentration of noradrenaline after clonidine

Treatment		Number	NA (ng/mg prot.)	NM (ng/mg prot.)	(NM)/(NA)
T1	T2				
Frontal cortex					
Saline	Saline	6	168 ± 11	11 ± 0.7	7 ± 0.4
Saline	CLO/0,1	8	215 ± 10*	8 ± 0.6*	4 ± 0.3*
1MeTIQ/25	Saline	6	182 ± 5	41 ± 1.8**	22 ± 0.7**
1MeTIQ/25	CLO/0,1	8	274 ± 21**^, +^	27 ± 2.7**^, ++^	11 ± 1.7*^, ++^
Effect of 1MeTIQ			*F*_(1, 23)_ = 5.62	*F*_(1, 23)_ = 169.18	*F*_(1, 23)_ = 107.40
			*P* < 0.02	*P* < 0.0000001	*P* < 0.0000001
Effect of CLO			*F*_(1, 23)_ = 20.21	*F*_(1, 23)_ = 17.68	*F*_(1, 23)_ = 42.02
			*P* < 0.0001	*P* < 0.0003	*P* < 0.000001
Interaction of 1MeTIQ + CLO			*F*_(1, 23)_ = 2.15	*F*_(1, 23)_ = 8.93	*F*_(1, 23)_ = 17.86
			ns	*P* < 0.006	*P* < 0.0003
Hypothalamus					
Saline	Saline	6	1194 ± 62	13 ± 0,8	1.2 ± 0.12
Saline	CLO/0,1	8	1407 ± 101	7 ± 0,9*	0.5 ± 0.05*
1MeTIQ/25	Saline	6	1366 ± 103	55 ± 3**^, ++^	4.0 ± 0.3**^, ++^
1MeTIQ/25	CLO/0,1	8	1475 ± 141	32 ± 3**^, ++^	2.1 ± 0.3**^, ++^
Effect of 1MeTIQ			*F*_(1, 23)_ = 1.075	*F*_(1, 23)_ = 226.4815	*F*_(1, 23)_ = 109.6038
			ns	*P* < 0.0000001	*P* < 0.0000001
Effect of CLO			*F*_(1, 23)_ = 1.9194	*F*_(1, 23)_ = 40.6956	*F*_(1, 23)_ = 25.2442
			ns	*P* < 0.000002	*P* < 0.000044
Interaction of 1MeTIQ + CLO			*F*_(1, 23)_ = 0.1971	*F*_(1, 23)_ = 15.0388	*F*_(1, 23)_ = 6.5234
			ns	*P* < 0.0007	*P* < 0.0177
Striatum					
Saline	Saline	6	29 ± 7	8 ± 0.8	26 ± 5
Saline	CLO/0,1	8	31 ± 5	5 ± 0.6*	16 ± 7*
1MeTIQ/25	Saline	6	41 ± 7*	14 ± 1.3*^, +^	33 ± 5*^, +^
1MeTIQ/25	CLO/0,1	8	40 ± 5*	11 ± 1.6	28 ± 3^+^
Effect of 1MeTIQ			*F*_(1, 23)_ = 6.705	*F*_(1, 23)_ = 12.162	*F*_(1, 23)_ = 0.141
			*P* < 0.01	*P* < 0.001	ns
Effect of CLO			*F*_(1, 23)_ = 0.370	*F*_(1, 23)_ = 4.312	*F*_(1, 23)_ = 4.094
			ns	*P* < 0.041	*P* < 0.050
Interaction of 1MeTIQ + CLO			*F*_(1, 23)_ = 0.180	*F*_(1, 23)_ = 2.232	*F*_(1, 23)_ = 2.945
			ns	ns	*P* = 0.050

1MeTIQ in a dose of 25 mg/kg i.p. was administered 30 min before clonidine (0.1 mg/kg i.p.; combined group). The control group received saline. Animals were decapitated 90 min after clonidine injection. The concentration of noradrenaline (NA) and its metabolite normetanephrine (NM) was expressed as nanograms per gram of wet tissue. The rate of NA metabolism was expressed as the ratio of its metabolite concentration to noradrenaline, (NM)/to (NA) × 100. The data are the means ± SEM. The results were analyzed by means of two-way ANOVA analysis of variance, followed when appropriate, by post hoc Duncan’s test

*P < 0.05; **P < 0.01 vs. control group; ^***+***^ *P* < 0.05; ^*++*^ *P* < 0.01 vs. clonidine-treated group—statistical significance

##### NM

One-way ANOVA demonstrated a significant but opposite effect of 1MeTIQ and clonidine as well as their interaction in all investigated structures: frontal cortex, hypothalamus, and striatum. Duncan’s post hoc test indicated that clonidine led to a significant decrease (from 35 to 50% of the control; *P* < 0.05) in NM level in all investigated brain structures. Also, 1MeTIQ induced an opposite effect to clonidine and significantly increased the NM level (from 60% of control, *P* < 0.05 in the striatum to about 300% of the control, *P* < 0.001 in the frontal cortex and hypothalamus) (Table 3).

##### The Index of Noradrenaline Catabolism

Two-way ANOVA indicated a significant but opposite effects of the treatments: 1MeTIQ and clonidine as well as their interaction 1MeTIQ + clonidine on the rate of noradrenaline catabolism (NM)/(NA). The effect of treatment with 1MeTIQ was significant in the frontal cortex (*F*_(1, 23)_ = 107.40, *P* < 0.00000) and hypothalamus (*F*_(1, 23)_ = 109.60, *P* < 0.00000) and that of clonidine in all investigated structures: frontal cortex (*F*_(1, 23)_ = 42.02, *P* < 0.00000), hypothalamus (*F*_(1, 23)_ = 25.24, *P* < 0.00004), and striatum (*F*_(1, 23)_ = 54.094, *P* < 0.05). Their interaction also revealed a statistical significance (frontal cortex: *F*_(1, 23)_ = 17.86, *P* < 0.0003; hypothalamus: *F*_(1, 23)_ = 6.52, *P* < 0.017; striatum: *F*_(1, 23)_ = 2.94, *P* < 0.05). Duncan’s post hoc test demonstrated a significant decrease (from 40%, *P* < 0.05 in the frontal cortex and striatum; about 50%, *P* < 0.05 in the hypothalamus) in the rate of noradrenaline catabolism after clonidine treatment. As opposed to clonidine, administration of 1MeTIQ led to a strong, significant increase (about 300%, *P* < 0.001) in the rate of noradrenaline catabolism in the frontal cortex and hypothalamus, and a weaker impact in the striatum (about 27% of control, *P* < 0.05) (Table 3).

#### The Effect of 1MeTIQ on Clonidine-Induced Depression Manifested by Changes in the Serotonin Metabolism

##### Serotonin

One-way ANOVA indicated a significant effect of treatment with 1MeTIQ on the level of serotonin in all investigated structures: frontal cortex, hypothalamus, and striatum (*F*_(1, 23)_ = 58.87, *P* < 0.00000; *F*_(1, 23)_ = 5.53, *P* < 0.027; *F*_(1, 23)_ = 9.89, *P* < 0.004, respectively) and no effect of clonidine treatment and interaction of 1MeTIQ with clonidine (Table 4). Duncan’s post hoc test indicated that administration of 1MeTIQ produced a significant increase in serotonin concentration in the frontal cortex (about 30% of control, *P* < 0.01) and in the hypothalamus (about 17% of control, *P* < 0.05) (Table 4).

**Table 4:** One-way ANOVA indicating a significant effect of treatment with 1MeTIQ on the level of serotonin in all investigated structures

Treatment		Number	5-HT (ng/mg prot.)	5-HIAA (ng/mg prot.)	(5-HIAA)/(5-HT)
T1	T2				
Frontal cortex					
Saline	Saline	6	522 ± 21	156 ± 6.9	30 ± 0.5
Saline	CLO/0,1	8	578 ± 14	131 ± 4.6**	23 ± 0.6**
1MeTIQ/25	Saline	5	678 ± 17**	109 ± 6.8**	17 ± 1.1**
1MeTIQ/25	CLO/0,1	8	765 ± 28**^, ++^	103 ± 3.9**^, ++^	13 ± 0.8**^, ++^
Effect of 1MeTIQ			*F*_(1, 23)_ = 58.87	*F*_(1, 23)_ = 50.46	*F*_(1, 23)_ = 215.43
			*P* < 0.0000001	*P* < 0.0000001	*P* < 0.0000001
Effect of CLO			*F*_(1, 23)_ = 3.22	*F*_(1, 23)_ = 9.50	*F*_(1, 23)_ = 36.95
			ns	*P* < 0.005	*P* < 0.000003
Interaction of 1MeTIQ + CLO			*F*_(1, 23)_ = 2.55	*F*_(1, 23)_ = 2.17	*F*_(1, 23)_ = 2.58
			ns	ns	ns
Hypothalamus					
Saline	Saline	6	924 ± 33	239 ± 11	26 ± 0.3
Saline	CLO/0,1	8	967 ± 40	188 ± 6**	20 ± 1**
1MeTIQ/25	Saline	5	1074 ± 59*	163 ± 10**^, +^	15 ± 1**^, ++^
1MeTIQ/25	CLO/0,1	8	1019 ± 37	100 ± 5**^, ++^	10 ± 1**^, ++^
Effect of 1MeTIQ			*F*_(1, 23)_ = 5.536	*F*_(1, 23)_ = 111.96	*F*_(1, 23)_ = 120.294
			*P* < 0.02755	*P* < 0.0000001	*P* < 0.0000001
Effect of CLO			*F*_(1, 23)_ = 0.019	*F*_(1, 23)_ = 53.517	*F*_(1, 23)_ = 38.516
			ns	*P* < 0.000001	*P* < 0.000002
Interaction of 1MeTIQ + CLO			*F*_(1, 23)_ = 1.306	*F*_(1, 23)_ = 0.615	*F*_(1, 23)_ = 0.176
			ns	ns	ns
Striatum					
Saline	Saline	6	405 ± 32	332 ± 20	83 ± 3.4
Saline	CLO/0,1	8	385 ± 9	290 ± 10*	75 ± 1.8*
1MeTIQ/25	Saline	5	456 ± 13	283 ± 18*	62 ± 2.8**
1MeTIQ/25	CLO/0,1	8	444 ± 11^+^	244 ± 11**^, +^	55 ± 2.1**^, ++^
Effect of 1MeTIQ			*F*_(1, 23)_ = 9.89	*F*_(1, 23)_ = 10.59	*F*_(1, 23)_ = 67.72
			*P* < 0.004	*P* < 0.003	*P* < 0.0000001
Effect of CLO			*F*_(1, 23)_ = 0.820	*F*_(1, 23)_ = 7.91	*F*_(1, 23)_ = 8.51
			ns	*P* < 0.009	*P* < 0.007
Interaction of 1MeTIQ + CLO			*F*_(1, 23)_ = 0.05	*F*_(1, 23)_ = 0.02	*F*_(1, 23)_ = 0.02
			ns	ns	ns

1MeTIQ in a dose of 25 mg/kg i.p. was administered 30 min before clonidine (0.1 mg/kg i.p.; combined group). The control group received saline. Animals were decapitated 90 min after clonidine injection. The concentration of serotonin (5-HT) and its metabolite, 5-hydroxyindolacetic acid (5-HIAA) was expressed as nanograms per gram of wet tissue. The rate of 5-HT metabolism was expressed as the ratio of its metabolite concentration to serotonin, (5-HIAA)/to (5-HT) × 100. The indices were calculated using concentrations from individual tissue samples. The data are the means ± SEM. The results were analyzed by means of two-way ANOVA analysis of variance, followed when appropriate, by post hoc Duncan’s test

*P < 0.05; **P < 0.01 vs. control group; ^***+***^ *P* < 0.05; ^*++*^ *P* < 0.01 vs. clonidine-treated group—statistical significance

##### 5-HIAA

One-way ANOVA demonstrated a significant effect of treatments: 1MeTIQ and clonidine on the level of 5-HIAA in the tested structures: frontal cortex (*F*_(1, 23)_ = 50.46, *P* < 0.000000; *F*_(1, 23)_ = 9.50, *P* < 0.005 respectively), hypothalamus (*F*_(1, 23)_ = 111.96, *P* < 0.00000; *F*_(1, 23)_ = 53.51, *P* < 0.000001, respectively), and striatum (*F*_(1, 23)_ = 10.59, *P* < 0.003; *F*_(1, 23)_ = 7.91, *P* < 0.009, respectively) and no significant effect of their co-administration. Duncan’s post hoc test indicated that both clonidine and 1MeTIQ significantly decreased the concentration of 5-HIAA in all investigated brain structures (Table 4).

##### The Index of Serotonin Catabolism

One-way ANOVA revealed a significant effect of treatments: 1MeTIQ and clonidine on the index of serotonin catabolism (5-HIAA)/(5-HT) in all analyzed structures: frontal cortex (*F*_(1, 23)_ = 215.43, *P* < 0.000000; *F*_(1, 23)_ = 36.95, *P* < 0.000003, respectively), hypothalamus (*F*_(1, 23)_ = 120.29, *P* < 0.00000; *F*_(1, 23)_ = 38.51, *P* < 0.000002, respectively), and striatum (*F*_(1, 23)_ = 67.72, *P* < 0.000000; *F*_(1, 23)_ = 8.51, *P* < 0.007, respectively) and no significant effect of their co-administration on the index of serotonin catabolism (Table 4).

## Discussion

Depression is a multicausal disorder which affects up to 25% of the worldwide population (Maes 2008). For the last two decades, the monoaminergic hypothesis of depression had been thought to explain the dominant cause of depression, and generally, antidepressant agents were produced based on this hypothesis. The MAO inhibitors, although not used as extensively as some other antidepressants, have an important role in the treatment of atypical depression and depression associated with anxiety, agitation, and phobia. However, other strategies beyond serotonin and noradrenaline reuptake inhibition such as anti-oxidant and anti-inflammatory agents may represent promising antidepressant therapeutics, which was already demonstrated by researchers in animal models of depression (Lopresti et al. 2012; Lang and Borgwardt 2013). Additionally, it should be taken into account that some natural compounds with antidepressant potential (e.g., curcumin), although their mechanism of the antidepressant activity is not fully understood, have been hypothesized to act through inhibiting the MAO enzyme and modulating the release of 5-HT and DA (Kulkarni et al. 2009). Similarly, the disturbances in the function of vesicular monoamine transporter 2 (VMAT2) in the cytosol which is responsible for the accumulation of biogenic amines in presynaptic stores may be the cause of the molecular processes which produce neurotoxicity and underlying depression disease (Miller et al. 1999).

1MeTIQ, an endogenous compound present in the mammalian brain exerts a distinct antidepressant-like effect in some animal models of depression (FST, reserpine model of depression) as it was previously demonstrated by us (Antkiewicz-Michaluk et al. 2014b; Możdżeń et al. 2014; Wąsik et al. 2013). What is interesting is that 1MeTIQ possesses antidepressant-like activity both in the behavioral as well as in neurochemical studies. Generally, its antidepressant-like effect was associated with the complex mechanism of action in the CNS. 1MeTIQ activates monoaminergic systems, elevates the concentrations of dopamine, noradrenaline, and serotonin in the brain structures as a reversible MAO inhibitor, and acts as a scavenger of free radicals.

In the present paper, we used clonidine as a useful and well-known animal model of depression (Enginar and Eroglu, 1990; Parale and Kulkarni 1986; Park et al. 2016) to investigate the antidepressant-like effect of 1MeTIQ. Clonidine is an alpha_2_ adrenoceptor agonist used in clinical practice in the therapy of hypertension; however, depression is known to be a side effect of clonidine treatment. Similarly, the induction of depression-like behavior in rats using clonidine was well documented (Dyr et al. 2009; Enginar and Eroglu 1990; Kostowski and Obersztyn 1988; Parale and Kulkarni 1986). In the present study, we demonstrated the antidepressant potential of 1MeTIQ in the clonidine model of depression. To begin the behavioral tests, it was demonstrated that clonidine used in a low dose (0.1 mg/kg i.p.) produced a distinct inhibitory effect on the locomotor activity, which was significantly antagonized by 1MeTIQ administration (Fig. 2). On the other hand, another behavioral test, i.e., the FST used in the present study indicated that clonidine did not change the antidepressant-like effect of 1MeTIQ connected with the direct shortening of the immobility time of rats; however, it completely prevented the 1MeTIQ-induced changes in the swimming behavior (Fig. 1a, B). In the joint treatment group (clonidine + 1MeTIQ), a significant higher climbing behavior was observed (Fig. 1c). What does it mean that in the FST, clonidine completely antagonized 1MeTIQ-induced swimming behavior? We suggest that such effect can be explained by the different mechanisms of action of clonidine and 1MeTIQ in relation to motivational processes. The FST is a behavioral test used to predict the efficacy of antidepressant treatments (Porsolt et al. 1977). It has a good predictive value for antidepressant potency in humans (Wilner 1984). The modified FST measures the frequency of different types of active behaviors: swimming, which is sensitive to serotoninergic compounds, such as specific serotonin reuptake inhibitors (SSRIs), and climbing, which is sensitive to tricyclic antidepressants and drugs with selective effects on catecholamine transmission (Cryan and Lucki 2000; Cryan et al. 2005; Detke et al. 1995, 1996). As shown by Detke et al. (1995, 1996), the increase in climbing activity is connected with an enhanced noradrenaline system activation. In that case, clonidine as an alpha_2_ adrenoreceptor agonist inhibits the activity of noradrenergic neurons and should break the climbing behavior. Such discrepancies in the clonidine mechanism of action can be connected with the function of the postsynaptic alpha_2_ adrenoceptors in the spinal cord which plays an essential role in locomotion. In fact, Giroux et al. (2001) demonstrated in the intact and spinal cuts that noradrenaline-descending system played a crucial role in the control of locomotion since stimulation of the noradrenergic postsynaptic alpha_2_ receptor by clonidine in the spinal cord prolonged the cycle duration and increased the amplitude of flexor and extensor bursts. In contrast, blockade of alpha_2_ receptor by intrathecal injection of yohimbine, its antagonist, resulted in a marked disruption of walking (Chau et al. 1998; Giroux et al. 2001). Clonidine may exert an effect primarily on interneurons that coordinate the timing between flexor and extensor muscle (Chau et al. 1998). In the light of these findings, it is possible that stimulation of the postsynaptic spinal noradrenergic alpha_2_ receptor by clonidine may be responsible for the increase in the climbing behavior observed in the FST.

We should also take into account that clonidine as an agonist of the presynaptic alpha_2_ noradrenergic receptors inhibits noradrenergic neurons in the locus coeruleus (LC) (Aghajanian and Vandermaelen 1982). In fact, the data from experimental animals suggested that all effective antidepressant drugs as well as electroconvulsive shock caused a decrease in the activity of LC neurons, the major noradrenaline-containing cell bodies (Grant and Weiss 2001; Bourin et al. 2002). Additionally, clonidine in low dose potentiated the anti-immobility effects of antidepressants in the FST in mice (Bourin et al. 2002; Zeidan et al. 2007).

It should be underlined that, except for the FST, other behavioral test (for example, locomotor activity test) and particularly biochemical studies (the inhibition of monoamine metabolism in the brain structures) indicate the “pro-depressive-like” effects produced by clonidine administration. It is well documented that monoamine neurotransmitters are involved in the pathogenesis of depression and play an important role in mediating the effects of antidepressants (Javaid et al. 1979; Borsini and Meli 1988). Both clinical and experimental data agree on the fact that classical antidepressants (imipramine and desipramine) as well as SSRI and SNRI activate monoaminergic neurotransmission in the brain (Grunewald et al. 1979; Javaid et al. 1979; Borsini and Meli 1988; Borsini 1995; Cryan and Lucki 2000; Cryan et al. 2005; Detke et al. 1995). Neurochemical data in the present paper demonstrated that clonidine produced a significant inhibition of monoamine neurons. The release of dopamine, noradrenaline, and serotonin as well as the rate of their metabolism was diminished in the investigated brain structures (frontal cortex, hypothalamus, and striatum; Tables 1, 2, 3, and 4). The indices presented in the paper such as (3-MT)/(DA), (3-MT)/(DOPAC), (NM)/(NA), and (5-HIAA)/(5-HT) have shown a distinct reduction of the activity of monoaminergic neurons in all investigated brain structures. 1MeTIQ which demonstrated a significant antidepressant-like effect in the FST completely antagonized clonidine-induced depression of monoaminergic systems and restored their function.

Based on comparison of the results of behavioral and biochemical experiments, we suggest that noradrenergic alpha_2_ receptors in the brain and in the spinal cord are different. It seems that spinal postsynaptic alpha_2_ receptors in contrast to brain presynaptic alpha_2_ receptors are not connected with the motivation function. In fact, 1MeTIQ did not antagonize the effect of clonidine on the climbing behavior in the FST but completely antagonized the clonidine-induced depression in the locomotor activity test as well as the reduction of brain monoaminergic neuron activity.

It should be mentioned that 1MeTIQ is characterized by a wide spectrum of actions on all monoaminergic systems in rat brain. Thanks to its ability to inhibit both MAO A and B activities and to scavenge free radicals, 1MeTIQ possesses a neuroprotective activity (Patsenka and Antkiewicz-Michaluk 2004; Antkiewicz-Michaluk et al. 2006). These results justify the question about the physiological significance of endogenous tetrahydroisoquinoline in the control of neurotransmitter function and prevention of neurotoxicity related to MAO activity in the brain. We would like to suggest that 1MeTIQ may be useful not only for the therapy of neurodegenerative diseases (e.g., PD) but also in the treatment of depression as a new antidepressant which can markedly reduce the side-effect profile.
